# Evidentiary discrepancies in sexual assault casework within the US

**DOI:** 10.1080/20961790.2021.1960465

**Published:** 2021-08-27

**Authors:** Chinyere M. Williams

**Affiliations:** Department of Chemistry and Forensics, Nottingham Trent University, Nottingham, UK

**Keywords:** Forensic sciences, forensic toxicology, drug-facilitated, sexual assault, DFSA, chemical submission, SANE, SART

## Abstract

In recent years, a significant number of investigations have discovered up to 200 000 unsubmitted sexual assault kits (SAKs) in the US. While the public outcry was largely directed towards DNA analysis, the SAKs also contained biological specimens specifically designated for toxicological analysis. Due to the sensitivity of analytes in potential drug facilitated sexual assaults, the preservation and maintenance of the specimens is crucial in providing accurate toxicological measurements. The investigations into the unsubmitted SAKs have identified subjective law enforcement officer (LEO) rationale for the unsubmitted kits, however the impact on toxicological specimens has not been examined. This brief review of policies and guidelines with respect to forensic specimens has identified potential sources of evidentiary degradation, despite the use of chemical preservatives. With respect to temperature-controlled environments, the variation in SAK submission policies established throughout the US are potentially detrimental to the preservation of toxicological evidence. Degradation as a result of time-delayed collection and poorly maintained storage temperatures plays a crucial role for/in the interpretation of qualitative and quantitative toxicological results. This review finds these delays can be addressed through modernisation of facilities; electronic tracking of unsubmitted SAKs; mandated transfer of biological evidence within 72 h; and documentation of temperature within the chain of custody or other records. Without identifying the range of temperatures in which the evidence was exposed, forensic toxicologists may unintentionally provide erroneous interpretations of toxicological analyses – potentially casting doubt on the survivor’s recall of events and negatively impacting future sexual assault investigations.

## Key points

- Temperature-controlled conditions for biological evidence of sexual assault cases may be inadequate in the US.
- Biological specimens collected in drug-facilitated sexual assault (DFSA) casework must be immediately preserved in optimal temperature-controlled temperatures.
- If biological specimens are not stored at optimal temperatures, forensic toxicologists are likely to interpret values that do not reflect the specimen at time of collection.
- It would benefit DFSA investigations and toxicological interpretations if temperature-related information was included with the chain of custody or other included documentation.

## Introduction

Expedient collection and analysis of toxicological evidence in forensic investigations is paramount. As a result, biological evidence collected from drug-facilitated sexual assaults (DFSAs) require temperature-controlled conditions or chemical additives to preserve potential drugs in the specimens. In the US, substantial delays in submission of DFSA evidence to forensic laboratories have been identified as a national issue [1–3]. These reports identified approximately 100 000–200 000 unsubmitted sexual assault kits (SAKs) due to a variety of procedural timelines [4, 5], however, much of the focus has been on the untested DNA evidence rather than the impact on toxicological evidence.

When a person survives a sexual assault incident, they may present to a hospital or rape treatment clinic for evaluation and/or examination. At this stage, they would be examined by trained professionals and interviewed by a sexual assault nurse examiner (SANE) and/or law enforcement officer (LEO). During the examination, potential evidence is collected and documented for further analysis. The SAK used by trained medical or forensic professionals provides all the tools necessary to safely collect and gather evidence following a sexual assault. Although the kit contents can vary between jurisdictions and municipalities [1], they typically include instructions for collection, swabs for DNA, vials for blood collection, sterile jars for urine collection, labels for evidence, bags for storage, and documentation to initiate a chain of custody [6, 7]. With respect to forensic toxicology, the results of the analyses performed on collected biological speci­mens do not necessarily “solve” a case, but they can support the perceived credibility of the survivor [8].

Incidents involving DFSA may be described in two ways: a “*proactive* DFSA” where a person is given drugs covertly or by force, and an “*opportunistic* DFSA” where a person is assaulted while they are incapacitated [9, 10]. In either scenario, the survivor may feel hesitant to seek medical treatment due to shame, embarrassment, and/or other personal factors [11]. In these instances, a critical amount of biological drug evidence may be lost to metabolic processes, causing analytical complications for mass spectral analysis. In addition to the described delays, the improper storage of biological specimens prior to submission for toxicological analysis may negatively impact the preservation of the evidence.

The accurate interpretation of drug concentrations in human-performance casework is critical to the field of clinical and forensic toxicology [12]. Degradation of evidence during handling could result in erroneous interpretations and have harmful legal consequences. The objectives of this paper are to identify potential sources of evidentiary degradation with respect to DFSA-related toxicology and qualitatively assess the impact of submission delays with respect to drugs associated with DFSA investigations.

## Methodology

Over a 2-month period, articles for this review were mined and identified using the following online databases and resources: Nottingham Trent University Library One Search, Google Scholar, Web of Science, and US Department of Justice. Using those resources, the following terms were searched: DFSA, chemical submission, toxicology stability, SAKs, toxicology guidelines, and unsubmitted SAK. Content that did not provide evidentiary procedures, statistical analyses, and clinical or forensic case studies were not included in this review. The articles used in this review paper were largely based in the US, however, other jurisdictions and international protocols were considered as well.

## Results

The detection and identification of drugs and alcohol in DFSA evidence is historically difficult. The reasons for this issue vary but are largely connected to the quick metabolism and degradation of drugs commonly used in assaults. According to previous reports [10, 12], the most effective drug to facilitate a sexual assault has several qualities: an ability to cause sedation or amnesia, have no odour or flavour, easy to conceal in a beverage, and, can be rapidly metabolised by the body. With these characteristics in mind a variety of drugs have been identified as toxicological candidates in DFSA investigations. At minimum, alcohol, amphetamines, benzodiazepines, cannabinoids, cocaine, opiates, and γ-hydroxy butyrate (GHB) should form the panel of tested drugs [9], though ketamine, barbiturates, chloral hydrate, antihistamines, and other synthetic analogues have been identified in case reports [13, 14].

Due to the presence of drugs and alcohol in DFSA incidents, survivors may report a loss of memory and/or consciousness during the event [13]. Depending on the amount of time elapsed between an assault and the examination of survivors at a medical facility, blood and/or urine may be selected for DFSA analysis. If the assault occurred within 72 h of the survivor’s examination, collection of both blood and urine are suggested [6, 9, 13]. For the collection of blood, sodium fluoride and potassium oxalate are required, whereas urine does not require such preservation [15]. Although chemical preservation is not required for long-term storage of urine, the stability of GHB is increased when urine is kept at −20 °C or contains sodium fluoride [16]. Once the specimens are collected, specific temperature conditions are required to preserve the specimens until they are ultimately analysed by the forensic laboratory.

In Table 1, recommended short-term storage conditions are listed. Short-term storage is described as less than 72 h while long-term storage is any time exceeding 72 h [7]. For storage of biological evidence, blood vials should be stored from 2 °C–8 °C and urine jars should be frozen at a temperature of at least −20 °C. Although the specimens may have preservative, the temperature-controlled environment is crucial in maintaining the quality of the specimens [16–20]. It is important to note that these recommendations would occur at the medical facility where the specimens were initially collected. For DFSA-related biological specimens, the responsibility of long-term storage resides with the law enforcement agencies or forensic laboratory performing the analyses [7].

**Table 1.** Short-term storage conditions for biological speci­mens of drug-facilitated sexual assault (DFSA) cases, based on information provided by US government documents [7, 15].

	Short-term storage conditions (°C)			
Evidence	<−20	2 – 8	15.5 – 24	Room temp.
Liquid blood	Never	Yes	<24 h	–
Urine	Yes	<24 h	–	–
Dry biological stain	–	–	Yes	Acceptable

Once the specimens are collected and submitted to LEO personnel, the biological evidence must be expediently transferred to the forensic laboratory for analysis. Over a 10-year period, surveys identified approximately 100 000–200 000 unsubmitted SAKs by LEO personnel, terminating any potential analysis by forensic laboratories [6, 7]. In fact, one survey found only 25% of total SAKs were submitted for analysis within 1 year of the collection date [2]. When surveyed, LEOs had a variety of reasons for not taking the prepared biological evidence to the forensic laboratories. In a survey conducted by Campbell and Fehler-Cabral [5], LEOs did not submit evidence because the forensic laboratory was unable to accommodate the long-term storage of evidence. In those situations, the LEOs had to make a decision on whether the case was “worth” pursuing. Other LEOs felt the credibility of the survivor was questionable and the assault was a false report [2, 4, 5]. Regardless of the reasons for the unsubmitted SAKs, the storage conditions of the biological specimens remain in question.

As seen in Table 2, no national consensus exists with respect to the time permitted between collection of biological evidence and the submission to a forensic laboratory [7]. Some LEOs described difficulties reaching state laboratories due to the lengthy travel times. Nevertheless, considering the information provided in Table 1 and the addition of nece­ssary preservatives, the biological evidence should experience minimal degradation during transport [7].

**Table 2.** Partial list of states in US and time requirements for submission of biological evidence for sexual assault cases, based on information provided by a government document [7].

State(s)	Collection to LEO	LEO to laboratory
**Florida,New Mexico, Texas, Washington, Idaho, Utah**	No requirement	Within 30 d
**Colorado**	No requirement	Within 21 d
**Illinois**	No requirement	Within 10 business days
**California**	No requirement	Within 20 d
**Connecticut**	No requirement	Within 10 d
**Ohio**	No requirement	Within 30 d for DNA tests
**Wisconsin**	No requirement	“In a timely manner”
Tennessee, Virginia	No requirement	Within 60 d for DNA
Arizona	Within 5 d	Within 15 business days
District of Columbia	Within 7 d	Within 7 d
Georgia	96 h	Within 30 d
Kentucky	Within 5 d	Within 30 d
Pennsylvania	Within 72 h	Within 15 d of consent for testing

LEO: law enforcement officer.

## Discussion

### Survivor experience

With the previous information in mind, it is impor­tant to acknowledge the unintentional delays during the transfer of evidence to the toxicological laboratory. In this review, there were several instances where the survivor requested their biological specimens to remain unsubmitted to forensic laboratories. A portion of surveyed survivors expressed a lack of desire to attend court where they may potentially see the suspect again or lose wages due to time constraints [21]. Survivors have also opted to have an examination or treatment for the assault but did not want to prosecute their assailants. In some instances, survivors did not want to admit they had been using drugs at the time of the incident and opted to forego a drug analysis. Additionally, the interactions between the survivor and SANE personnel are identified as critical inflection points where a survivor decides to pursue an eventual prose­cution [22]. Further to this point, survivors reported they were unlikely to report an assault in the future if they had a negative interaction with SANE or LEO personnel [10]. Keeping in mind the rapid metabolism and degradation of potential DFSA-related drugs, any hesitation on the part of the survivor can have a significant impact on the drugs ultimately detected by the forensic laboratory.

According to an investigation by Leder [13], a sexual assault exam should occur within 72 h after the incident, although other investigations have found the deterioration of GHB, flunitrazepam, and ethanol in 12 h following the assault [9]. Given a scenario where a survivor consumed incapacitating drugs, there is an increased likelihood of additional delays with respect to collection of the biological specimens, further reducing the concentration of DFSA-related compounds [14]. In hospital facilities which do not have sufficient SANE personnel, survivors of assaults are often asked to wait for several hours in a waiting room prior to receiving treatment [22]. This scenario, in combination with the possible discomfort from reporting the incident, can further diminish the possibility of detecting xenobiotics. As noted by Hindmarch et al. [23], the highest percentage of positive cases occurred when the specimen was collected within 24 h of the incident. Any further delays or improper handling of the biological evidence may potentially result in inadequate or negative toxicological results.

### LEO involvement

Considering the intoxicating and incapacitating effects of drugs associated with DFSAs, the delays permitted in Table 2 are of great concern with respect to toxicological interpretations. While it is reasonable to expect analytical delays when LEOs are required to travel long distances in order to submit biological evidence, the length of time the SAK specimens are held in storage prior to transfer may be detrimental. Based on previously described literature, the storage conditions required to preserve biological specimens may exceed the abilities of a typical law enforcement division. The acquisition of freezers which maintain temperatures of −20 °C or less can have a significant fiscal impact. In addition to the financial investment, the long-term storage of biological specimens potentially requires physical changes to a facility. LEOs are unlikely to have facilities which can accommodate such requirements.

In an investigation by Muldoon et al. [24], DFSA cases led by Canadian law enforcement revealed similarly low submission rates. In their investigation, only 60/202 of SAKs were submitted for forensic analysis over the specified period. In contrast to some jurisdictions in the US, the survivor of a ­se­xual assault must provide explicit consent for the SAK to be released to the police. Ideally, law enforcement agencies and forensic laboratories would utilise the same procedures and techniques when investigating DFSA casework, however neither the SAKs nor the departmental practises are standardised in the US and other parts of the world [1, 9]. As a result of the report by the US Department of Justice, 27 states have required LEOs to submit all completed SAKs to forensic laboratories, however the contents of the kits are not yet standardised [1]. To date, there are jurisdictions who propose establishing an electronic tracking system of all generated SAKs, providing the law enforcement agencies and forensic laboratories a more robust method of auditing unsubmitted casework.

### Specimen handling

Due to the logistical difficulties presented from standardisation of analytical procedures, laboratories may consider taking a more judicious approach with respect to specimen handling and documentation. With respect to specimen temperature, the short- and long-term storage temperatures could be docu­mented on the chain of custody, allowing the reviewing toxicologist to consider degradation as a result of suboptimal conditions. Though preserved with sodium fluoride and potassium oxalate, whole blood specimens require temperature-controlled environments. As shown in Shan et al. [25], blood ethanolic measurements can decrease as much as 0.02 g/dL if specimens are stored between the range of 4 °C–24 °C. Flunitrazepam, a benzodiazepine which causes anterograde amnesia, may be stable at −20 °C for up to 1 month, however the metabolite, 7-amino flunitrazepam, is unstable at −20 °C and requires temperatures of at least −60 °C for long-term maintenance [17, 20]. Again, the preservation of GHB in urine may be dependent on the addition of fluoride preservatives and storage at −20 °C. An investigation led by Wang et al. [6] found 492 of 868 sexual assault investigations occurring in San Francisco involved the loss of memory during the assault. In fact, some reports suggest the presence of GHB and ethanol are largely underreported based on the symptoms communicated by survivors of the assault to SANE personnel [9].

### Analytical procedures

In an investigation by Peters and Remane [26], limited sample preparation was determined to create significant matrix effects – negatively impacting the ability to discern the presence of drugs in low concentrations. Considering the low concentrations of drugs expended in a specimen collected several hours after an incident, this limited approach could reduce the possibility of identifying such compounds. Furthermore, as the biological specimens age without the proper temperature controls there could be an additive impact on the mass-spectral suppression or enhancement. Again, knowing the symptoms expressed by the survivor and identifying the freeze-thaw conditions the specimens can allow a toxicologist to interpret a realistic scenario in which metabolism and degradation are considered.

### Potential consequences of non-disclosure

Due to the nature of the crime, prioritising the concerns and wellness of DFSA survivors is of the upmost importance. If the final toxicology results do not explain or reflect the survivors’ experience, they may experience self-doubt and shame for pursuing prosecution. Additionally, the survivors may question and doubt their own memories of the assault, causing further distress. This negative experience may be shared within the local community, resulting in a shared distrust of the clinical and toxicological evaluations. Another consequence of poorly maintained biological evidence could be the effect on legal processes. As described by Campbell et al. [4], prosecution of DFSA cases is infrequent throughout the US. Those who adjudicate DFSA cases may find the toxicological results insubstantial and difficult to prosecute, making the survivor less likely to testify or participate in the process. With respect to the field of forensic toxicology, these discrepancies could lead to accusations of evidence mishandling, further calling into question the abili­ties of the laboratory.

### Proposed corrections

As the issue of unsubmitted SAKs became public, a collection of investigative reports have identified ways to address the backlog of casework [27]. As stated previously, the transition to electronic recordkeeping is believed to be an ideal way to track and monitor the storage conditions of unsubmitted SAKs [7]. While mandating the transfer of all SAK evidence to a forensic laboratory is ideal, an additional time constraint should be considered to ensure speci­mens are in suboptimal conditions for limited periods. Although modernisation and standardisation of forensic procedures can address these evidentiary shortfalls, the fiscal impact to law enforcement agencies and laboratories cannot be ignored. The use of national grants and other bursaries may help agencies develop practices that preserve all biological specimens – not solely for DFSA specimens. In addition to these proposals, there appears to be a need for additional training for LEOs and forensic laboratories regarding time-sensitive evidence submission.

Another area that impacts the detection of drugs is the application of chromatographic methods which employ large number of analytes. Forensic laboratories frequently have high caseloads which are ma­naged through the employment of analytical techniques detecting multiple drugs of interest. Although this is desirable for processing large numbers of cases, limiting the numbers of analytes in a liquid-chromatography mass-spectrometry (LC-MS) method can reduce ion suppression and enhancement [26]. This reduction in combination with extractions that take advantage of unique chemical properties of the analytes, such as light sensitivity, may increase the sensitivity of matrices that were stored in sub-optimal conditions. With existing guidelines regarding analytical method validation and matrix effects [15, 26], the establishment of analyte stability during freeze-thaw cycles and suboptimal long-term storage should be considered during the ultimate interpretation of results. Additionally, the use of enzymatic hydrolysis provides a measurable increase in the detection of protein-bound drugs while reducing the matrix effects commonly encountered in mass spectral ana­lyses [15, 26]. With respect to less commonly seen drugs such as synthetic cathinones [28], these adjustments to analytical procedures could greatly increase the scope and sensitivity of the methods. If hydrolytic procedures are not in place, it would be prudent to include glucuronide conjugates as part of the analytical array. Alternatively, the use of other biological matrices such as hair and blood spots may be preferred specimen types in jurisdictions where significant delays in analysis are unavoi­dable [16].

## Conclusion

Reduced analyte stability as a result of exposure to uncontrolled temperatures is a common occurrence in the US and other parts of the world. While this situation is not uncommon, it is impor­tant for forensic toxicologists to acknowledge the potential impacts of these environments on the reliability of the analytical results. As stated by Peters [17], “Reliable qualitative and quantitative toxicological analysis is the basis of a competent toxicological judgment and consultation in clinical and forensic toxicology. Unreliable results may lead to overestimation or underestimation of effects, to false interpretations, and to unwarranted conclusions. In the worst case, this might result in unjustified legal consequences for the defendant or wrong treatment of the patient”. To address the lack of transparency regarding the handling of the DFSA specimens, it would be prudent of forensic toxicologists to clearly delineate the potential loss of DFSA analytes when offering interpretations for legal proceedings.
